# Body composition in term offspring after maternal gestational diabetes does not predict postnatal hypoglycemia

**DOI:** 10.1186/s12887-021-02578-3

**Published:** 2021-03-06

**Authors:** Cornelia Wiechers, Lena S. Balles, Sara Kirchhof, Romy Weber, Vanessa Avellina, Jan Pauluschke-Fröhlich, Manfred Hallschmid, Louise Fritsche, Hubert Preißl, Andreas Fritsche, Christian F. Poets, Axel R. Franz

**Affiliations:** 1 Department of Neonatology, University Children’s Hospital, Eberhard Karls University, Calwerstr. 7, 72076 Tuebingen, Germany; 2 Department of Obstetrics and Gynecology, University Hospital, Eberhard Karls University, Calwerstraße 7, 72076 Tübingen, Germany; 3 Institute for Medical Psychology and Behavioural Neurobiology, Eberhard Karls University, Otfried-Müller-Straße 25, 72076 Tübingen, Germany; 4 German Center for Diabetes Research, Eberhard Karls University, Tuebingen, Germany; 5 Institute for Diabetes Research and Metabolic Diseases of the Helmholtz Center Munich at the Eberhard Karls University, Tuebingen, Germany; 6 Department of Internal Medicine IV, Eberhard Karls University, Tuebingen, Germany; 7 Center for Pediatric Clinical Studies, University Children’s Hospital, Eberhard Karls University, Tuebingen, Germany

**Keywords:** Infant, Gestational diabetes, Fetal hyperinsulinemia, Neonatal, Body composition, Air displacement plethysmography, Fat mass

## Abstract

**Background:**

Offspring of mothers with gestational diabetes mellitus (GDM) have an increased risk of neonatal complications like birth trauma due to macrosomia or postnatal hypoglycemia, as well as long-term metabolic sequelae. Neonatal body composition may be a sensitive marker of metabolic effects on the fetus caused by suboptimal glycemic control during pregnancy.

**Objective:**

To determine body composition in offspring of mothers with GDM compared to a reference cohort of healthy term neonates and to assess whether increased body fat would be associated with postnatal hypoglycemia.

**Methods:**

This prospective, observational, cross-sectional study included 311 full-term, singleton infants born between June 2014 and July 2015. Body composition was measured within 96 h of birth using air displacement plethysmography. Results are indicated as median (1st Quartile – 3rd Quartile).

**Results:**

Of 311 infants, 40 (12.9%) were born to mothers with GDM. Birth weight standard deviation scores (SDS) (0.24 vs. − 0.07, *p* = 0.04), fat mass (370 g vs. 333 g, *p* = 0.02) as well as fat mass/total body mass (BF%; 11.4% vs. 10.8%, *p* = 0.03) were significantly higher in infants following maternal GDM than in controls. In GDM offspring, anthropometric parameters, fat mass or BF% did not differ between infants with or without postnatal hypoglycemia. In this cohort, SDS for birth weight, fat mass, fat free mass, BF% or postnatal hypoglycemia were not associated with maternal blood glucose levels measured at an oral glucose tolerance test.

**Conclusions:**

SDS for birth weight, neonatal fat mass, and BF% were significantly higher in newborns following maternal GDM. In these infants born to mothers with GDM, body composition did not differ between those with or without postnatal hypoglycemia.

## Background

The prevalence of gestational diabetes mellitus (GDM) is increasing worldwide [1]. Currently, up to 17% of pregnant women are affected, with prevalence varying widely (2–25%) depending on nationality, screening methods and diagnostic threshold [2–5]. The 1-year prevalence of GDM in Germany, based on outpatient data for the nationwide introduction of GDM screening, is at 13.2% and thus in the range seen internationally [3]. Offspring of mothers with GDM have an increased risk of adverse perinatal outcomes (e.g., makrosomia, birth injury, respiratory distress syndrome and postnatal hypoglycemia) and long-term consequences including type II diabetes and metabolic syndrome in adulthood [6–8]. The underlying pathomechanism is still not completely understood.

The Hyperglycemia and Adverse Pregnancy Outcome (HAPO) Study enrolling 23,316 participants showed strong continuous associations between maternal glucose levels and increased birth weight or cord-blood C-peptide levels [6, 9]. These findings seem to confirm the Pedersen hypothesis, which postulated that maternal hyperglycemia is transferred to the fetus, causing an exaggerated fetal insulin response [10]. The resulting fetal hyperinsulinemia is thought to cause various aspects of diabetic fetopathy, including the deposition of large amounts of body fat. After birth, infants born to mothers with elevated glucose levels during pregnancy have a high risk of postnatal hypoglycemia because glucose supply via the umbilical cord is immediately interrupted, while neonatal insulin levels are still elevated. Newborn hypoglycemia is associated with long-term neurologic impairment [11, 12], infants born to mothers with GMD are therefore screened to prevent postnatal hypoglycemia [13, 14].

Neonatal adiposity may be an indicator of poor glycemic control during pregnancy, however, birth weight or body mass index (BMI) at birth correlate poorly with neonatal adiposity as indicated by considerable variability in neonatal body composition parameters such as fat mass (FM), lean mass (FFM) and the proportion of FM divided by total body mass (BF%) in neonates of similar weight and length [15, 16]. Thus, FM and BF% might be more sensitive markers of the uterine environment and better indicators for the risk of postnatal hypoglycemia or adverse metabolic sequelae in later life than anthropometric parameter alone [17].

The aim of this study was to determine body composition in infants of mothers with and without GDM using air displacement plethysmography (ADP) soon after birth and to assess, whether BF% or FM are associated with postnatal hypoglycemia. We used ADP as this is the gold standard for measuring neonatal body composition, is fast, non-invasive and without ionizing radiation, making it suitable for large epidemiological studies with high reproducibility and accuracy [18, 19].

## Methods

### Participants

This was a prospective cross-sectional study of term, singleton infants (≥37 0/7 weeks of pregnancy) born between June 2014 and July 2015 at Tuebingen University Women’s and Children’s Hospital, Germany. Infants were recruited by the study team on the maternity ward if they fulfilled inclusion criteria (singleton, gestational age at birth ≥37 + 0/7 weeks), preferably on the day after birth, to enable measurements at the latest 96 h after birth. The aim was to address as many parents as possible, but recruitment was restricted by limited availability of the study team. Infants with major congenital anomalies (e.g., congenital heart defects, diaphragmatic hernia, and chromosomal aberrations) or severe disease (e.g., severe perinatal acidosis, meconium aspiration syndrome) were excluded. Recruited neonates were divided by pregnancy history into a healthy reference group and those with evidence of maternal GDM as identified by the national GDM screening program.

In Germany, a nationwide screening for GDM was introduced in 2012 for all pregnant women. In week 24–28 of pregnancy, a screening test involving oral administration of 50 g glucose is performed and, if the capillary glucose level exceeds 135 mg/dl (7.5 mmol/l) after 1 hour, followed by an oral glucose tolerance test (oGTT; 75 glucose, fasting for at least 8 h). The diagnostic criteria for GDM applied herein are based on those from the International Association of Diabetes and Pregnancy Study Group (IADPSG) [20] and defines GDM as present if the following blood glucose thresholds are exceeded in the 75-oGTT: fasting, 92 mg/dl (5.1 mmol/l), 1 h, 180 mg/dl (10.0 mmol/l) or 2 h, 153 mg/dl (8.5 mmol/l).

Maternal pre-pregnancy body mass index (BMI) (in kg/m^2^) was calculated as pre-pregnancy weight divided by height squared. The following BMI categories were used: underweight (BMI < 18.5), normal weight (18.5–24.9), overweight (25.0–29.9) and obese (> 30) [21]. The Institute of Medicine (IOM) recommendations concerning gestational weight gain for singleton pregnancies depending on the maternal pre-pregnancy BMI were used to classify weight gain during pregnancy: underweight (recommended gestational weight gain: 12.5–18.0 kg); normal weight (11.5–16.0 kg), overweight (7.0–11.5 kg) and obese (5.0–9.0 kg) [21]. Gestational weight gain below, within or above the recommended range according to maternal pre-pregnancy BMI was classified as “insufficient”, “adequate” and “excessive”, respectively.

In infants born to mothers with prenatally diagnosed GDM, capillary blood glucose measurements were taken at birth and 1, 3, 6, 9 and 12 h after birth according to institutional guidelines, these measurements were subsequently stopped if blood glucose was > 45 mg/dl (2.5 mmol/l) throughout. In addition, newborns were breastfed early and frequently and were given supplementary feeds with a milk protein-free energy supplement 8x10ml per day (Aptamil Primergen®, Milupa, Friedrichsdorf). Neonatal hypoglycemia was defined as a blood glucose level < 45 mg/dl (2.5 mmol/l).

### Ethics

The Institutional Review Board approved the study protocol and written informed parental consent was obtained. This trial was initiated prior to the ICMJE requirement for trial registration of observational studies.

### Clinical data collection

Data were collected from maternal health passports and blood glucose diaries as well as maternal and neonatal medical records; parents were also asked to fill in a questionnaire. Medical data included age, pre-pregnancy BMI, parity, gestational weight gain, smoking during pregnancy, antenatal medical history and, if a GDM was detected, its date of diagnosis and treatment (e.g., insulin, diet). Neonatal data included age, sex, anthropometric parameters at birth and at the time of body composition measurement (weight, length, head circumferences) and blood glucose values where applicable.

### Anthropometric measures and body composition

Body composition was measured with ADP using PeaPod® Infant body composition system (COSMED®, Rome, Italy), where BF%, FM and FFM were calculated by determining weight and body volume [18, 22]. Neonatal anthropometric measures and body composition were performed within 96 h of birth. Body mass was measured to the nearest 0.1 g using the digital scales of the PeaPod®, length to the nearest 0.1 cm using a recumbent, digital infant length board (Ulmer Stadiometer, Busse, Ulm, Germany) and head circumference to the nearest 1 mm using a non-stretchable tape measure.

### Area under the curve of maternal blood glucose levels during 75 g oGTT

As indicator of “severity” of maternal gestational diabetes, maternal blood glucose levels (BGlc) measured at 75 g oGTT were converted into an area under the curve (AUC) as follows: AUC 75 g oGTT in [min*mg/dl] = ((60 min*BGlc_fasting_) + 1/2*60 min*(BGlc_1hr_ - BGlc_fasting_) + 1/2*60 min*(BGlc_1hr_ - BGlc_2hr_) + 60*(BGlc_2hr_)).

### Calculation of standard deviation scores (SDS) for weight, length and head circumference

These parameters were computed using LMSgrowth (version 2.14; http://www.healthforallchildren.com/?product=lmsgrowth). The reference population was the British 1990 growth reference (23, 24) fitted by maximum penalized likelihood as described before [23].

### Statistical analyses

Data are presented as mean and (standard deviation (SD)) if normally distributed, or as median and interquartile range (1st Quartile – 3rd Quartile) if not. In case that within a table a minority of parameters were normally distributed, all data in that table were presented as median (Q1-Q3) to improve clarity of presentation. Between-group comparisons were performed using two-sided t-test or Wilcoxon test for non-normally distributed data and Fisher’s exact test for categorical outcomes. To assess correlations between AUC for maternal blood glucose levels during 75 oGTT and birth weight SDS, fat mass and BF% were assessed by linear regression and Pearson correlation coefficients were calculated. Analyses were performed with GraphPad Prism® 8.1.0 (GraphPad Software, San Diego, CA, USA) and the level of significance was *p* < 0.05.

## Results

### Participants

There were 3170 deliveries at Tuebingen University Women’s Hospital during the one-year recruitment period; measurements of body composition using ADP could be performed in 311 healthy infants with gestational age > 37 weeks. These included a reference cohort of 271 healthy, singleton term infants of GDM-free mothers (already published previously [25]) and 40 infants of mothers with GDM, based on the documented values of the 75 g glucose tolerance test.

#### Maternal characteristics

Mothers with GMD had a significantly higher pre-pregnancy weight, pre-pregnancy BMI and were more often overweight or obese (BMI > 25 kg/m^2^) compared to the reference cohort.

In the reference cohort, no mother reported that she had been diagnosed with GDM. In some pregnant women from the reference cohort, a 75 g oGTT was performed directly instead of only after a GDM screening test; thus, blood glucose concentrations of the 75 g oGGT were available for 64 women in this cohort. Detailed daily blood glucose profiles were also not available in all pregnant women with GDM, therefore, the quality of glycemic control cannot be determined.

#### Infant characteristics

SDS birth weight in infants born to mothers with GDM was significantly higher than in the reference cohort (SDS birth weight 0.24 and − 0.07, respectively, *p* = 0.04d). There was no significant difference in anthropometric parameters between infants born during the study period and not included in the study and the reference study or GDM cohort. Demographic data of the study population are shown in Table 1.

**Table 1:** Characteristics of newborn and maternal demographic data and infants body composition

		Study cohort GDM ***n*** = 40	Reference cohort ***n*** = 271	All singleton infants > 37 weeks ***n*** = 2225^**a**^
**Infant characteristics at birth**				
Female	n (%)	18 (45%)	153 (57%)	1099 (49%)
Gestational age at birth (weeks)	Median (Q1, Q3)	39.7 (38.6–40.3)	39.9 (39.0–40.4)	39.7 (38.9–40.4)
SDS Birth weight	Median (Q1, Q3)	0.24*^#^ (− 0.24–0,86)	-0.07 (−0.62–0.59)	− 0.05 (− 0.65–0.59)
Birth weight (g)	Median (Q1, Q3)	3520 (3188–3760)	3420 (3050–3675)	3370 (3080–3680)
Birth length (cm)	Median (Q1, Q3)	52 (50–53)	51 (50–52)	51 (50–52)
Birth head circumference (cm)	Median (Q1, Q3)	35 (34–37)	35 (34–36)	35.0 (34–36)
**Maternal characteristics**				
Age at delivery (years)	Median (Q1, Q3)	33.9 (29.0–38.8)	32.6 (29.2–35.9)	
Pre-pregnancy weight (kg)	Median (Q1, Q3)	75* (66–84)	63 (57–72)	
Pre-pregnancy BMI (kg/m^2^)	Median (Q1, Q3)	26.7 * (23.7–31.6)	22.5 (20.6–25.2)	
Pre-pregnancy BMI categories	n (%)			
Underweight (BMI < 18.5)		2 (5%)^b^	7 (3%)	
Normal weight (BMI 18.5–24.9)		11 (28%)^b^	193 (71%)	
Overweight (BMI 25–30)		15 (38%)^b^	46 (17%)	
Obese (BMI > 30)		12 (30%)^b^	25 (9%)	
Gestational weight gain (kg)	Median (Q1, Q3)	12.0 (8.0–17.6)	14.0 (11.0–18.0)	
Gestational weight gain categories^d^	n (%)			
Insufficient		10 (25%)	55 (20%)	
Adequate		9 (23%)	100 (37%)	
Excessive		21 (53%)	116 (43%)	
Nulliparous	n (%)	19 (48%)	149 (55%)	
Parity	Mean (SD)	1.7 (0.8)	1.6 (0.8)	
Vaginal Delivery	n (%)	22 (55%)	175 (64%)	
Pregnancy-induced hypertension	n (%)	6 (15%)*	7 (3%)	
Familial predisposition for hypertension or diabetes mellitus	n (%)	16 (40%)*	45 (17%)	
Plasma glucose 75 g oGTT (mg/dl) Fasting	Median (Q1, Q3)	94* (91–99)	79^c^ (75–84)	
1 h		174* (143–185)	125 ^c^ (107–145)	
2 h		142* (107–171)	100 ^c^ (88–111)	
AUC (mg/dl*min)		17340* (15255–18,731)	12,930 (11610–14,378)	
Therapy of GDM	n (%)		–	
No therapy		6 (15%)		
Diet		20 (50%)		
Insulin therapy		14 (35%)		
**Infants’ Characteristics at measurement**				
Postnatal age (h)	Median (Q1, Q3)	42.5 (34.3–52.3)	42.0 (29.2–56.0)	
Weight loss since birth (g)	Median (Q1, Q3)	179 (114–244)	185 (132–247)	
Weight (g)	Median (Q1, Q3)	3332 (3042–3619)	3218 (2887–3488)	
**Infants’ body composition**				
Fat free mass (g)	Median (Q1, Q3)	2909 (2669–3148)	2843 (2606–3099)	
BF (%)	Median (Q1, Q3)	11.4* (9.7–15.0)	10.8 (7.7–13.4)	
Fat mass (g)	Median (Q1, Q3)	370* (286–512)	333 (226–443)	

*Abbreviations*: *BMI* Body mass index, *GDM* gestational diabetes mellitus, *SD* standard deviation, *oGTT* oral glucose tolerance test

*GDM cohort significantly different from reference cohort [25]

#SDS birth weight in all singleton, healthy term infants > 37 wk. born in recruitment period and not recruited to this study was significantly different from GDM cohort (*p* = 0.05)

^a^ All singleton, healthy, term infants > 37 weeks born in the recruitment period at Tübingen Women’s Hospital excluding study participants *n* = 2225. Data retrospectively extracted without identifiers from the hospital quality assurance database

^b^ GDM cohort significantly different from reference cohort (underweight/normal vs. overweight/obese, Fishers’s exact test, *p* < 0.0001)

^c^ In the reference group, results of the 75 g oGTT were available in 64 cases

^d^ Gestational weight gain categories, classified according to the Institute of Medicine recommendations 2009 taking the pre-pregnancy BMI into account

#### Association of maternal GDM with infant body composition

In infants of mothers with GDM, BF% (11.4% vs 10.8%, *p* = 0.030) and fat mass (370 g vs. 333 g, *p* = 0.022) was significantly higher than in the reference cohort (Table 1). In boys born to mothers with GDM, FFM was significantly higher than in girls (3094 g vs. 2823 g, *p* = 0.036). Birth weight, SDS birth weight, BF% and fat mass were not different in both sex in the GDM cohort (Table 2). In boys born to mothers with GDM, SDS birth weight, BF%, and FM were significantly higher compared to boys in the reference cohort.

**Table 2:** Subgroup analysis in female and male infants of GDM study population

		GDM cohort		Reference cohort	
		Female	Male	Female	Male
**Infants characteristics**					
Birth weight (g)	Median (Q1, Q3)	3370 (2863–3780)	**3637**^**a**^ (3408–3750)	3320 ^c^ (2990–3600)	3520 (3130–3798)
SDS Birth weight	Median (Q1, Q3)	0.32 (−0.56–0.72)	0.17 (− 0.15–0.92)	−0.04 (− 0.57–0.65)	−0.07 (− 0.70–0.44)
**Infants’ body composition**					
Fat mass (g)	Median (Q1, Q3)	371 (288–542)	**365**^**a**^ (296–481)	347 (239–446)	303 (219–438)
Fat mass / total body mass (%)	Median (Q1, Q3)	11.8 (9.9–15.4)	**11.3**^**a**^ (9.3–14.1)	11.2 ^c^ (8.7–14.0)	9.6 (7.2–12.1)
Fat-free mass (g)	Median (Q1, Q3)	2823 (2515–2948)	**3094**^**b**^ (2892–3242)	2768 ^c^ (2541–3021)	2977 (2714–3154)

^a^Male infants in GDM cohort significantly different from male infants in reference cohort [25]

^b^Male infants in GDM cohort significantly different from female infants in GDM cohort

^c^Male infants in reference cohort significantly different from female infants in reference cohort [25]

#### Neonatal body composition and postnatal hypoglycemia

In total, at least one hypoglycemic episode with a blood glucose level < 45 mg/dl (2.5 mmol/l) was documented in 17/35 (49%) newborns of mothers with GDM during the first 24 postnatal hours, the age at the last measured hypoglycemia was 1 h (1 h–6 h) and the number of documented hypoglycemic blood glucose results was 1 [1, 2]. Newborns of mothers with GDM who had developed hypoglycemia were not significantly different regarding sex, birth weight (g), SDS birth weight, fat mass or BF% from those without hypoglycemia (Table 3).

**Table 3:** Subgroup analysis infants of GDM study population with or without neonatal hypoglycemia

		Study cohort GDM No Hypoglycemia ***n*** = 18	Study cohort GDM ≥1 Hypoglycemia ***n*** = 17	***p***
**Maternal characteristics**				
Pre-pregnancy BMI (kg/m^2^)	Median (Q1, Q3)	26.0 (24.3–27.2)	29.4 (24.1–34.0)	0.10
Gestational weight gain categories^a^	n (%)			0.47
Insufficient		4 (22%)	6 (35%)	
Adequate		6 (33%)	2 (12%)	
Excessive		8 (44%)	9 (53%)	
Maternal insulin therapy	n (%)	7 (39%)	7 (41%)	1.0
**Infant characteristics**				
Female	n (%)	12 (67%)	6 (35%)	0.09
Birth weight (g)	Median (Q1, Q3)	3555 (2998–3878)	3530 (3340–3750)	0.93
SDS birth weight	Median (Q1, Q3)	0.28 (−0.33–0.90)	0.38 (−0.31–0.83)	1.0
Blood glucose postnatal (mg/dl)	Median (Q1, Q3)			
0-1 h		67 (58–87)	49 (37–67)	0.02
1-3 h		65 (58–68)	48 (40–63)	0.05
3-6 h		65 (58–70)	64 (51–70)	0.60
6-12 h		60 (58–67)	49 (43–66)	0.04
12-24 h		69 (60–74)	55 (52–63)	0.04
Minimal blood glucose level	Median (Q1, Q3)	55 (52–59)	36 (32–40)	< 0.0001
**Infants’ body composition**				
Fat mass (g)	Median (Q1, Q3)	377 (280–582)	380 (350–444)	0.73
Fat mass / total body mass (%)	Median (Q1, Q3)	12.5 (9.3–16.0)	11.3 (10.1–13.8)	0.44
Fat-free mass (g)	Median (Q1, Q3)	2891 (2535–3127)	2996 (2678–3251)	0.46

*Abbreviations*: *BMI* body mass index, *GDM* gestational diabetes mellitus, *SDS* standard deviation score, *oGTT* oral glucose tolerance test

^a^Gestational weight gain categories, classified according to the Institute of Medicine recommendations 2009 taking the pre-pregnancy BMI into account

#### Relationship between maternal glucose levels during 75 g oGTT and infant outcomes

**Fig. 1:**
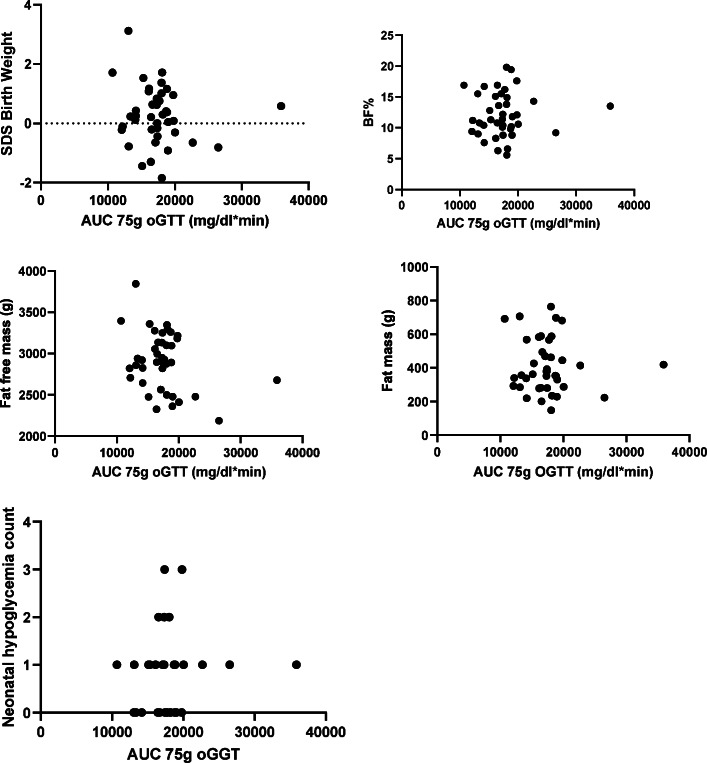
Scatter plot AUC 75g oGTT vs. SDS birth weight, BF%, FFM, FM and neonatal hypoglycemia count (GDM cohort)

SDS birth weight, neonatal hypoglycemia count or BF% of infants born to mothers with GDM were not associated with the AUC of 75 g oGGT, see Fig. 1.

## Discussion

The aim of this cross-sectional observational study was to determine the difference in body composition in healthy singletons born to mothers with GDM compared to a reference cohort of neonates born to mothers without GDM. Furthermore, we wanted to investigate whether postnatal hypoglycemia in newborns of mothers with GDM is associated with neonatal body composition, since an elevated BF% could serve as a surrogate marker for suboptimal glycemic control during pregnancy.

Significant differences were found in fat mass (370 g vs. 333 g, *p* = 0.02) and BF% (11.4% vs. 10.8%, *p* = 0.03) between infants of mothers with GMD compared to the reference cohort. These differences were statistically significant, but mean differences were small and probably of little clinical relevance. We are aiming to re-examine these children’s anthropometrics and body composition in subsequent years to verify whether the small difference at birth translates into clinically relevant differences later in life. In a meta-analysis of 10 studies using different body composition techniques (six studies used skinfold thickness, three ADP and one total body electrical conductivity), infants of mothers with all types of diabetes mellitus during pregnancy were found to have 83 g or 22% more pooled fat mass and 2.2% higher BF% after birth compared to infants of mothers with normal glucose tolerance [26]. In contrast, in a cross-sectional study reporting good glycemic control in about 90% of 67 participating women with GDM, there was also no difference in neonatal BF% compared to a reference cohort [27]. Our GDM cohort seems to be in between – with significantly higher BF% (but smaller mean difference of 0.6%) and higher fat mass (mean difference 37 g). The offspring of mothers with diabetes have a higher risk of adverse metabolic health later in life, and obesity can be a plausible mediator. In a recently published meta-analysis of five studies with a total of 890 children, the offspring of GDM mothers from prepubertal age to early adulthood showed higher 2-h plasma glucose levels in weight-appropriate oGTTs compared to controls [8]. Therefore, adequate treatment of GDM may be effective in preventing neonatal adiposity and may possibly also have a positive effect on long-term metabolic outcome.

In our GDM cohort, there were no associations between birth weight, SDS birth weight/BF% and postnatal hypoglycemia. We had hoped that increased BF% could be used as a predictor for postnatal hypoglycemia in infants born to diabetic mothers, to enable better identification of those at greatest risk. Overall, infants born to mothers with all types of diabetes mellitus during pregnancy have a high risk of neonatal hypoglycemia [6, 13, 14]. In our study population, about half the infants born to mothers with GDM were diagnosed with hypoglycemia < 45 mg/dl (< 2.5 mmol/l) in the first 24 h after birth. This is consistent with the literature, where up to 50% of term infants developed severe hypoglycemia with blood glucose levels below 36 mg/dl (< 2 mmol/l) in the first hours after birth, even in well-controlled diabetic mothers [13, 14]. Severe and recurrent hypoglycemia in newborns is associated with neurologic impairment, but there is little evidence to support any given threshold for intervention [11, 12]. In a prospective cohort study involving 528 at-risk term and late-preterm infants, neonatal hypoglycemia with a treatment threshold of 47 mg/dl (2.6 mmol/l) was not associated with adverse neurodevelopmental outcomes at 2 years or increased risk of combined neurosensory impairment in a follow up at 4.5 years [11, 28]. However, at age 4.5 years, hypoglycemia was associated with a dose-dependent increased risk of poor executive and visual motor function [11].

In our GDM cohort, SDS birth weight, BF%, and the number of hypoglycemic episodes were not associated with area under the curve of blood glucose levels following a standard 75 g oGTT. In a prospective study of 50 women with GDM, the fasting value of 100 g oGTT correlated with FM and BF% of their infants, who had their body composition measured via total body electrical conductivity [29]. The HAPO study demonstrated that clinical neonatal hypoglycemia showed continuous linear associations with 1 and 2 h plasma glucose levels and the fasting plasma glucose level [9].

Sex is known to be an important determinant of body weight and body composition at birth and also throughout life [19, 30, 31]. Baby girls have lower birth weights but higher BF%, and girls and adult women also have a higher BF% and lower FFM than their male counterparts [32, 33]. The influence of elevated blood glucose levels in women with GDM also seems to have sex-specific influences on their offspring [34, 35]. Increased maternal fasting blood glucose levels were the major predictor of neonatal adiposity in male infants born to women with GDM, but had little effect on BF% in girls in a prospective study with 84 mother-child pairs [34]. Similarly, male school-age offspring of mothers with GDM at a mean age of 95 months showed higher adiposity than offspring of normoglycemic mothers; but this correlation could not be established for girls [35]. Maternal pre-pregnancy BMI was the primary predictor of obesity in female newborns but not in males [34].

In our reference cohort, a significantly higher median BF% and lower FFM was found in girls compared to boys. In newborns of mothers with GDM, BF% was not different between boys and girls. Girls’ body composition in the GDM and reference cohort did not differ, but the BF% and FM in the GDM cohort were significantly higher in boys than in the reference cohort. More boys were found in the group with hypoglycemia, but this was not significant.

As previously published, BF% and FM were significantly associated with maternal BMI before pregnancy in our reference cohort [25]. In agreement with this observation within the reference cohort, mothers with GDM had a higher pre-pregnancy BMI and their infants had higher BF% than those in the reference cohort. In a meta-analysis with a total of 26,509 children, offspring of mothers with GDM had higher BMI Z-scores in childhood, which was no longer significant after adjustment to the mother’s BMI before pregnancy [8], indicating a collinearity between maternal GDM status and maternal pre-pregnancy BMI. The association of neonatal body composition parameters with pre-pregnancy BMI and GDM reflects the impact of the intrauterine environment on offspring adiposity and potentially the future risk of metabolic syndrome. Therefore, neonatal body composition parameters, e.g., BF% may serve as surrogate endpoint for trials aiming at improving neonatal outcome through better glycemic control in obese mothers or those with GDM or a timely diagnosis of GDM and strict glycemic control.

A strength of this study is its rather large sample size of the reference cohort, which was representative of all newborns born during the study period. That 12.6% of mothers had GDM is within the expected range for Germany [3]. A limitation is that newborns were recruited on the maternity ward, thereby excluding infants born to mothers with GDM who were transferred to a neonatal unit. Furthermore, our results may be an effect of well-controlled GDM rather than an observation of how inadequately controlled GDM influences body composition at birth and the risk of postnatal hypoglycemia.

Additional information, e.g., detailed daily blood glucose profiles, maternal food intake or physical activity during pregnancy would have been helpful in retrospect to identify further important factors related to body composition at birth.

## Conclusions

This cross-sectional study demonstrates that singleton neonates from mothers with GDM who were managed on the maternity ward had increased BF% values compared to healthy term controls. Newborns of mothers with GDM who suffered from hypoglycemic episodes showed no significant differences in body composition compared to those without hypoglycemia, but this finding may be biased by recruitment occurring only on the maternity ward, thereby excluding infants needing i.v. dextrose. Furthermore, the incidence of hypoglycemia was not associated with the AUC of maternal glucose levels during the 75 g oGTT. Continued monitoring of body composition in this cohort of offspring of mothers with GDM into child- and adulthood is desirable to investigate long-term effects.

## Data Availability

De-identified individual data will not be made available, because trial subjects have not been asked to consent.

## Abbreviations

ADP: Air displacement plethysmography
BMI: Body mass index
BF%: Proportion of fat mass/total body
FM: Fat mass
FFM: Fat free mass
GDM: Gestational diabetes mellitus
oGTT: Oral glucose tolerance test
SD: Standard deviation
SDS: Standard deviation score
